# Cytoprotective Compounds Interfere with the Nutraceutical Potential of Bread Supplemented with Green Coffee Beans

**DOI:** 10.3390/antiox8070228

**Published:** 2019-07-19

**Authors:** Urszula Gawlik-Dziki, Marcin Luty, Dariusz Dziki, Michał Świeca, Katarzyna Piwowarczyk, Urszula Złotek, Jarosław Czyż

**Affiliations:** 1 Department of Biochemistry and Food Chemistry, University of Life Sciences, Skromna Str. 8, 20-704 Lublin, Poland; 2 Department of Cell Biology, Faculty of Biophysics, Biochemistry and Biotechnology, Jagiellonian University, Gronostajowa 7, 30-378 Kraków, Poland; 3 Department of Thermal Technology and Food Process Engineering, University of Life Sciences, Doświadczalna Str. 44, 20-280 Lublin, Poland

**Keywords:** coffee beans, wholemeal bread, prostate cancer, gastrointestinal digestion, cytoprotection

## Abstract

The proliferation and motile activity of prostate epithelial (Pnt2) and cancer cells (DU-145; PC-3) in the presence of bioavailable compounds from green coffee beans (GCB), wholemeal wheat bread (WMWB), and its GCB-fortified variant were analyzed. The considerable cytostatic and anti-invasive activity of GCB extracts was correlated with its phenolic contents. WMWB extract contained significantly lower levels of phenolics but still displayed relatively high cytostatic activity. However, the cytostatic properties of WMWB compounds were hardly augmented by 3% GCB flour supplementation. The cytoprotective activity of the WMWB compounds exerts a negative impact on the cytostatic activity of GCB compounds. These data confirm the relatively high chemopreventive potential of GCB. However, they also indicate that subtle interactions between bioavailable compounds in GCB and WMWB can negatively affect the nutraceutic potential of the fortified bread. Apparently, gastrointestinal processing differentially regulates the availability of individual compounds and affects the balance between the cytostatic and cytoprotective activity of the whole product. Our data show that comprehensive research is necessary before the fortification of a specific carrier with a specific supplement can be recommended.

## 1. Introduction

Coffee beans are commonly used as a raw material for hot drinks and beverages. Numerous bioactive compounds in green coffee beans (GCB) may account for their normalizing effect on human metabolism [1]. Accordingly, flour made from GCB is considered a promising food fortifier. It can enrich the fortified products with a spectrum of phenolic compounds [2,3,4,5,6]. Phenolics represent a diverse group of secondary plant metabolites. In particular, GCB phenolic compounds exhibit antioxidant, antimutagenic, and anti-in. flammatory properties in vitro. These properties result from the antiradical and reducing potential of the compounds and from their ability to modify the activity of kinases, phosphates, and pro-oxidative enzymes [5]. Consequently, the antioxidative properties of phenolics are responsible for their interference with intracellular oxidative stress and reactive oxygen species (ROS)–dependent signaling pathways, thus accounting for their cytoprotective activity. Recently, the fortification of bread products has been proposed as a method of daily diet enrichment with phenolic compounds. These facts prompted us to speculate about the introduction of GCB-fortified bread into the daily diet [7]. 

Attempts to introduce GCB phenolics in the daily human diet require in-depth knowledge on the cell-specific consequences of their bioactivity [1,8]. They cannot be estimated without careful examination of the effects of GCB phenolics on the physiology of normal and cancer cells. We have previously shown that the addition of GCB flour into wholemeal wheat bread (WMWB) increases the amounts of bioaccessible and bioavailable phenolics in the product. It also enhanced its antioxidative potency [6]. Furthermore, the fortification of white bread with broccoli sprouts increased its chemopreventive potential. This was illustrated by the prominent cytostatic response of stomach cancer cells to extracts from a broccoli-sprout-supplemented product. In the same experimental model, onion skins considerably augmented the phenolic content and cytostatic activity of white bread [9]. The beneficial effects of bread fortification are determined by numerous factors (including the quality and quantity of the fortifier), by the additive and subtractive effects of the activities of bread and GCB phenolics, and by the collective reactivity of multicellular systems to these compounds. This justifies intensified research on the interactions between GCB and WMWB compounds and on their consequences in vivo and in vitro. 

GCB phenolics are potentially bioaccessible and bioavailable in vitro; however, the interactions between these compounds and the bread matrix can differentially affect their release during gastrointestinal processing [6,10]. These interactions affect the bioavailability of individual compounds and their relative content, thus having a potential impact on the bioactivity of the whole product. This may be crucial for the chemopreventive activity of the product. According to a recent meta-analysis, the relative risk of prostate cancer in regular coffee drinkers was considerably reduced as compared to that in coffee non-drinkers [11,12]. Therefore, we focused on the chemopreventive potential of GCB-supplemented bread against prostate cancer in terms of the bioavailability of its compounds. We analyzed the proliferation and motility of prostate cancer (DU-145; PC-3) and epithelial (Pnt2) cells in the presence of extracts from GCB flour, raw WMWB, and from a GCB-fortified product. Furthermore, we compared the cytostatic, anti-invasive, and cytoprotective activities of the extract subjected to simulated mastication, gastrointestinal digestion, and absorption in vitro. Such an approach enabled us to address the possible consequences of bread fortification on the anti-cancer bioactivity of the product in terms of the bioavailability of GCB and WMWB phenolics. 

## 2. Materials and Methods 

### 2.1. Gastrointestinal Processing and Preparation of Extracts 

#### Plant Materials

Green coffee (*Coffea arabica*) beans were received from Cofeina Romuald Zalewski, Marki, Poland. Flour from green coffee beans (moisture content 11.5 mg/100 g) was prepared according to Dziki et al., 2015 [4]. Wholemeal wheat flour (WF) was purchased from a local supermarket in Lublin, Poland (protein, ash, and moisture contents: 14 mg/100 g, 1.89 mg/100 g, and 13.6 mg/100 g, respectively).

### 2.2. Bread Preparation

The control bread (C) was prepared from WF. For GC1, GC2, GC3, GC4, and GC5 production, the flour was replaced with GCB flour at levels of 1 g/100 g, 2 g/100 g, 3 g/100 g, 4 g/100 g, 5 g/100 g, respectively. In addition, 6 g of instant yeast and 12 g of salt were used. 

The amount of water necessary for the preparation of the dough was determined using a Farinograph (Brabender, Duisburg, Germany). After fermentation, the pieces of dough were divided (300 g) and baked in an electric oven (Sveba Dahlen, Fristad Sweden) at 230 °C for 30 min. After baking, the bread was stored at room temperature for 1 h, packed in polypropylene bags and stored at 21 °C for 24 h, freeze-dried, and comminuted [4].

### 2.3. Gastrointestinal Processing and Preparation of Extracts 

The buffer extract and extracts after simulated digestion and absorption were prepared according to Minekus et al. [13] with some modifications [14]. 

### 2.4. Cell Cultures and Medium Supplementation 

Human prostate cancer DU-145 (HTB-81) and PC3 cells (CRL-1435, both from American Type Culture Colection (ATCC, Manassas, VA, USA) were routinely cultivated in Dulbecco's-Modified Eagle's medium-F12 Ham (DMEM-F12) medium supplemented with 10% fetal bovine serum (FBS) and antibiotics (gentamycin, Krka d.d., Novo Mesto, Slovenia) in standard conditions (37 °C, 5% CO_2_) [15]. Epithelioid PNT2 prostate cells (Sigma-Aldrich St. Louis, MO, USA, cat. No. 95012613) were cultured in RPMI medium supplemented with 10% FBS. For endpoint experiments, cells were harvested upon confluence with 0.25% trypsin in PBS (Sigma-Aldrich; [16]), and the cells were seeded into culture dishes at the densities given below and incubated for 24 hours. Afterwards, the culture medium was replaced with a new one supplemented with the relevant extract. Lyophilized extracts from saline buffer masticated (SB), gastrointestinally digested (GD), and gastrointestinally digested/absorbed (GDA) products were dissolved in the medium to reach final extract concentrations equivalent to 150 and 250 g of the product per 75 kg of body mass. 

### 2.5. Cell Proliferation 

For cell proliferation tests, the cells were harvested with trypsinization and seeded into 12-well plates in culture medium supplemented with 10% FBS (Corning, NY, USA) at an initial density of 5.5 × 10^3^ cells/cm^2^. Then, the cells were incubated for 24 h at 37 °C before the medium was replaced with a fresh one without (vehicle control) or with the relevant extract (applied at the concentration given in the text). After 72 h of incubation, the cells were harvested by trypsinization and counted using a Coulter Z2 Beckman counter. Three independent experiments were performed for each condition [17].

### 2.6. Cell Motility

For the analyses of cell movement, the cells were seeded into 12–well plates (Corning) at a density of 10^4^ cells/cm². After 24 h, the medium was replaced with new medium without (control) or with the relevant extract applied at the concentration given in the text. Time-lapse recordings of cell movement were performed after 72 h incubation of the cells in the presence of the extracts at 37 °C and in 5% CO_2_ atmosphere using an inverted Leica DMI6000B microscope equipped with IMC optics and a cooled digital DFC360FX CCD camera. The cell trajectories were constructed on the basis of 72 centroid positions recorded over 480 minutes at 5 min time intervals using a dry 10×, NA-0.75 objective. They were presented in circular diagrams with the starting point of each trajectory situated at the plot center. For each data point measured, the trajectories of 50 cells were analyzed. The total length of the cell trajectory (µm) and total length of cell displacement (i.e., the distance from the starting point directly to the cell’s final position; µm) were quantified using the Hiro program (written by W. Czapla) as described [17,18]. Three independent experiments were performed for each condition.

### 2.7. Phenolics Content 

The total phenols content (TPC) was estimated according to Singleton and Rossi [19]. The amount of total phenolics was expressed as gallic acid equivalent (GAE) per gram of dry weight (DW) [mgGAE/g DW]. The values in the plots represent theoretical concentrations of individual compounds in the culture medium for the extract dilution equivalent to 250 g of product per 75 kg. 

### 2.8. Analysis of Caffeine and Phenolic acids

Samples were analyzed using a Varian ProStar high-performance liquid chromatography (HPLC) system separation module (Varian, Palo Alto, CA, USA) equipped with a Varian ChromSpher C18 reverse-phase column (250 mm, 4.6 mm) and ProStar diode-array detector DAD detector according to a previously described method [20]. Liquid Chromatography Electrospray Ionization Tandem Mass Spectrometric LC-ESI-MS/MS analysis of phenolic acids was performed as previously described [21].

### 2.9. Xanthine Oxidase Inhibition 

Xanthine oxidase inhibition (XOI) activities were estimated according to Sweeney, Wyllie, Shalliker, and Markham, 2001 [22]. Xanthine was used as the XO substrate. Inhibitory activity is expressed as efficient concentration -IC50: the sample concentration (mg DW/ml) required to obtain 50% activity.

### 2.10. Statistics 

Experimental data are presented as means ± standard deviation (SD) for biochemical analyses and means ± standard error of the mean (SEM) for anticancer activity assays. In biochemical analyses, statistical significance was estimated with Tukey’s test (for the data obtained from three independent samples of each extract in three parallel experiments; *n* = 9). For the estimation of the effect of extracts on cell proliferation, the results from three independent experiments performed in triplicate (*n* = 9) were subjected to statistical analyses using Mann-Whitney’s *U*-test. For the estimation of the effect of extracts on cell motility, the trajectories of 50 cells from three independent experiments (*n* = 50) were subjected to statistical analyses using Mann-Whitney’s *U*-test. Unless stated otherwise, the statistical tests were performed at a significance level of 0.05. Statistical tests were performed using Statistica 6.0 software (StatSoft, Inc., Tulsa, Oklahoma, USA). 

## 3. Results and Discussion

### 3.1. Bioavailable GCB Compounds Exert Cytostatic Effects on Prostate Cancer Cells

**Figure 1:**
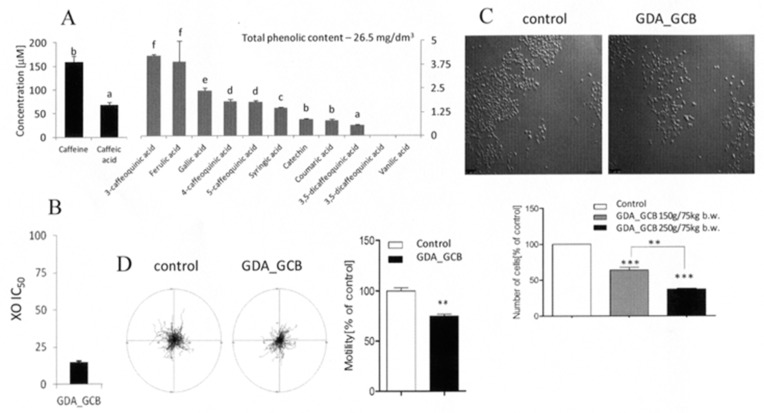
Bioavailable compounds in green coffee beans (GCB) exert cytostatic effects on prostate cancer DU145 cells. (**A**) GCB were subjected to simulated gastrointestinal digestion/absorption in vitro (see Section 2), and the extract was applied to the culture medium at a concentration equivalent to 250 g of product per 75 kg of body weight. Bar graphs show theoretical medium concentrations of the compounds calculated on the basis of their content in the extract. (**B**) GCB were processed as above, and the antioxidative and xanthine oxidase (XO) inhibitory activity of the extracts was estimated. (**C**) DU145 cells were seeded at the density of 10^4^/cm^2^ and cultivated for 24 h before gastrointestinally digested/absorbed (GDA) GCB extracts were administered along with 10% fetal bovine serum (FBS)-supplemented culture medium. Cell proliferation was measured after 72 h of incubation with a Coulter counter and compared to the control (= 100%) Scale bar: 100 μm. (**D**) DU145 cells were seeded at the density of 10^4^/cm^2^, then cultivated for 24 h and treated with GDE GCB extract for 72 h, and cell motility was analyzed via time-lapse videomicroscopy. Cell trajectories are presented in circular diagrams with the starting point of each trajectory situated at the plot center (*N* = 50). The bar graph shows averaged cell motility. All results are representative of at least three independent experiments performed in triplicate (*N* > 9). The values designated by the different letters (A) are statistically significantly different. *p* values in B and C were calculated using the non-parametric Mann–Whitney test, **p ≤ 0.001, **p ≤ 0.001. Note the cytostatic and anti-invasive effect of bioavailable GCB compounds on DU145 cells.

A multi-step protocol based on the simulated mastication (SB), gastrointestinal digestion (GD), and absorption (GDA) of fortified foods in vitro imitates their digestive processing and the differential penetration of natural biological barriers by their compounds. Their biological activity in cellular systems can be assessed after the administration of SB, GD, and GDA extracts to cell culture media at physiologically relevant concentrations. In this study, we applied the extracts at the concentrations equivalent to 150 and 250 g of the fortified product per 75 kg of human body weight (for details, see Section 2). The GDA_GCB-supplemented medium contained relatively high levels of phenolics, including caffeic, ferulic, and caffeoquinic acids and caffeine (Figure 1A). It was also characterized by considerable antioxidative activity and an inhibitory effect of the extract on xanthine oxidase (XO) activity [21] (Figure 1B). Redox- and XO-dependent signaling pathways are crucial for effective cell proliferation and motility. To estimate the potential of GCB for the chemoprevention of prostate carcinogenesis, we further analyzed the proliferation and motility of human prostate DU145 cells in the presence of gastrointestinally digested and absorbed (GDA) GCB extract.

Strong, dose-dependent inhibition of DU145 proliferation was observed in the presence of GDA_GCB extract (Figure 1C). This effect was accompanied by the inhibition of DU145 motility (Figure 1D). Previously, a corresponding cytoprotective effect of green coffee was observed in a human HepG2 cell model [23]. Our data suggest relatively strong anti-carcinogenic potential of the bioavailable GCB compounds. They indicate that GCB can be applied for prostate cancer chemoprevention, which justifies their implementation in the daily diet of humans. 

### 3.2. GCB Supplementation Slightly Affects the Cytostatic Properties of Bioavailable WMWB Compounds 

**Figure 2:**
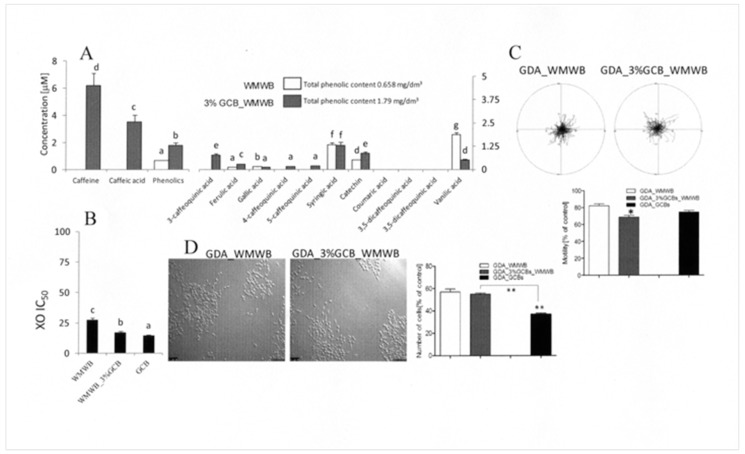
Bioactivity of bioavailable phenolics from raw wholemeal wheat bread (WMWB) and from its variant fortified with 3% GCB flour. (**A**) Raw and 3% GCB_WMWB were subjected to simulated gastrointestinal digestion/absorption in vitro, and the extract was applied to the culture medium at a concentration equivalent to 250 g of product per 75 kg of body weight. Bar graphs show the theoretical medium concentrations of the compounds calculated on the basis of their content in the extract. (**B**) GCB were processed as above and the antioxidative and XO inhibitory activity of the extracts was estimated. (**C**) DU145 cells were seeded at a density of 10^4^/cm^2^, cultivated for 24 h, and treated with GDE GCB extract for 72 h, and cell motility was analyzed via time-lapse videomicroscopy. Cell trajectories are presented in circular diagrams with the starting point of each trajectory situated at the plot center (*N* = 50). The bar graph shows averaged cell motility. (**D**) DU145 cells were seeded at a density of 10^4^/cm^2^ and cultivated for 24 h before GDA extracts of WMWB and of its fortified variant were administered along with 10% fetal bovine serum (FBS)-supplemented culture medium. Cell proliferation was measured after 72 h of incubation with a Coulter counter and compared to the control (= 100%). Scale bar: 100 μm. All results are representative of at least three independent experiments performed in triplicate (*N* > 9). The values designated by the different letters (A and B) are statistically significantly different. *p* values in C and D were calculated using the non-parametric Mann–Whitney test; *p ≤ 0.05, **p ≤ 0.01. Note the similar magnitude of cytostatic effects exerted by bioavailable WMWB and 3%C_WMWB compounds.

Fortification with GCB flour is an effective way of improving the pro-healthy properties of WMWB and increasing the daily dietary intake of GCB compounds, as previously shown for broccoli sprouts and onion skins [9]. Actually, WMWB does not contain bioavailable caffeine, caffeoquinic acids, or caffeic acid. Among the tested phenolics, the presence of syringic acid, vanillic acid, and (+) catechin was detected in the GDA_WMWB extract. These compounds reached concentrations between 0.4 and 1.2 μM in the supplemented culture medium (Figure 2A). The bioavailable WMWB compounds displayed relatively high antioxidative potential and a negligible inhibitory effect on xanthine oxidase (XO) activity (Figure 2B). Not surprisingly, the relatively low phenolic content of GDA_WMWB extract was considerably enhanced upon the fortification of WMWB with 3% GCB flour. GCB supplementation considerably increased the total content of bioavailable phenolics and augmented the antioxidative activity of the GDA_3%C_WMWB extract. For instance, the concentrations of caffeine, caffeic acid, caffeoquinic acid, and ferulic acid in the culture medium supplemented with this extract reached ca. 0.1–10% of the corresponding values estimated for the medium supplemented with GDA_GCB extract. Therefore, it was surprising to see that the inhibitory effects of bioavailable WMWB compounds on DU145 motility (Figure 2C) and proliferation (Figure 2D) were only slightly affected by 3% GDA supplementation. On the other hand, the activity of the GCB flour compounds was apparently retained in the fortified product, as illustrated by the enhanced anti-invasive activity of the GDA_3%C_WMWB medium (Figure 2C). 

Considerable bioactivity of WMWB phenolics remains, in contrast to our previous observations on the low phenolic content and bioactivity of white bread [24]. However, the lack of correlation between the bioactivity of the fortified product and the composition/abundance of its bioavailable compounds may also result from the relatively low concentration of GCB flour. Consequently, the amounts of bioactive GCB compounds were too low to effectively contribute to the relatively high cytostatic activity of WMWB compounds. On the other hand, the final inhibitory effect of the supplemented product on DU145 proliferation and motility depends on a subtle interplay between the cytostatic and cytoprotective activities of single WMWB and GCB compounds [9,24]. Accordingly, the cytostatic potential of GCB compounds can be counteracted by the cytoprotective activities of compounds donated by WMWB or vice versa. Furthermore, food processing can lead to shifts in the bioavailability of cytostatic/cytoprotective GCB and WMWB ingredients and to the nonlinearity of cellular reactions to the extracts [25].

### 3.3. Bioaccessible WMWB Compounds Interfere with Cytostatic GCB Compounds

**Figure 3:**
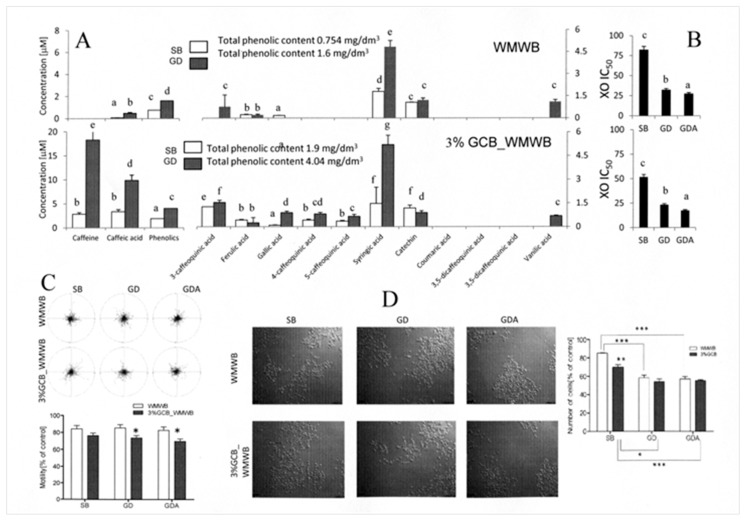
Gastrointestinal fate of compounds from WMWB and from its variant fortified with 3% GCB flour. (**A**) Raw and 3% GCB_WMWB were subjected to simulated mastication (SB) and gastrointestinal digestion (GD) in vitro, and the extract was applied to culture medium at a concentration equivalent to 250 g of product per 75 kg of body weight. The bar graphs show theoretical medium concentrations of the compounds calculated on the basis of their content in the extract. (**B**) GCB were processed as above, and the antioxidative and XO inhibitory activity of the extracts was estimated. (**C**) DU145 cells were seeded at a density of 10^4^/cm^2^, cultivated for 24 h, and treated with the relevant extract for 72 h, and cell motility was analyzed via time-lapse videomicroscopy. Cell trajectories are presented in circular diagrams with the starting point of each trajectory situated at the plot center (*N* = 50). The bar graph shows the averaged cell motility. (**D**) DU145 cells were seeded at a density of 10^4^/cm^2^ and cultivated for 24 h before SB and GD extracts of WMWB and of its fortified variant were administered along with 10% fetal bovine serum (FBS)-supplemented culture medium. Cell proliferation was measured after 72 h of incubation using a Coulter counter and compared to the control (=100%). Scale bar: 100 μm. All results are representative of at least three independent experiments performed in triplicate (*N* > 9). The values designated by the different letters (A and B) are statistically significantly different. *p*-values in C and D were calculated using the non-parametric Mann–Whitney test; *p ≤ 0.05, **p ≤ 0.01, ***p ≤ 0.001.Note the differences between the bioactivity of the SB and GD WMWB extracts and the lack of such differences between GD extracts from raw WMWB and its GCB-fortified variant.

To estimate whether gastrointestinal processing can affect the cytoprotective activity of WMWB compounds, we further compared the phenolic contents and bioactivity of media supplemented with SB/GD extracts from WMWB and 3%C_WMWB. Analyses of the “gastrointestinal fate” of WMWB compounds demonstrated that mastication released relatively small amounts of WMWB phenolics (Figure 3A), which resulted in low antioxidative activity of its SB extract (Figure 3B). Gastrointestinal digestion of WMWB released only a small fraction of phenolics, including 3-caffeoquinic, syringic, and vanillic acid; it also slightly increased its inhibitory effect on XO (Figure 3B) and augmented the cytostatic activity of WMWB extract (Figure 3C). These data suggest that the gastrointestinal processing of WMWB somehow eliminates cytoprotective WMWB phenolics rather than releasing their cytostatic counterparts. Consequently, the activity of cytostatic compounds is no longer balanced by the activity of their cytoprotective counterparts. This notion was further confirmed by the analyses of the phenolic content and cytostatic activity of the media supplemented with SB and GD 3%C_WMWB extracts. Gastrointestinal processing considerably increased the phenolic content of 3%C_WMWB extract (Figure 3A). Concomitantly, gastrointestinal processing increased the XO inhibitory activity (Figure 3B) and cytostatic properties of GD 3%C_WMWB extract (Figure 3C), without a significant impact on its anti-invasive activity (Figure 3D). Despite the significant differences in phenolic content observed upon the processing of both extracts, their cytostatic and antiinvasive effects remained similar. These observations indicate that the bioaccessible (GD-extractable) compounds of WMWB do not affect the activity of their cytostatic counterparts but can protect DU145 cells from the cytostatic action of GCB compounds. Because they are released from the bread matrix by gastrointestinal digestion and efficiently penetrate simulated intestine barriers in vitro, they can counteract the cytostatic activity of the bioavailable compounds donated by the fortifier [24,25,26]. Thus, our data confirm the role of synergic/antagonistic interactions between the cytostatic and cytoprotective compounds of WMWB and GCB in determining the fortified product’s bioactivity.

### 3.4. Gastrointestinal Digestion Attenuates the Activity of Cytoprotective GCB Compounds 

**Figure 4:**
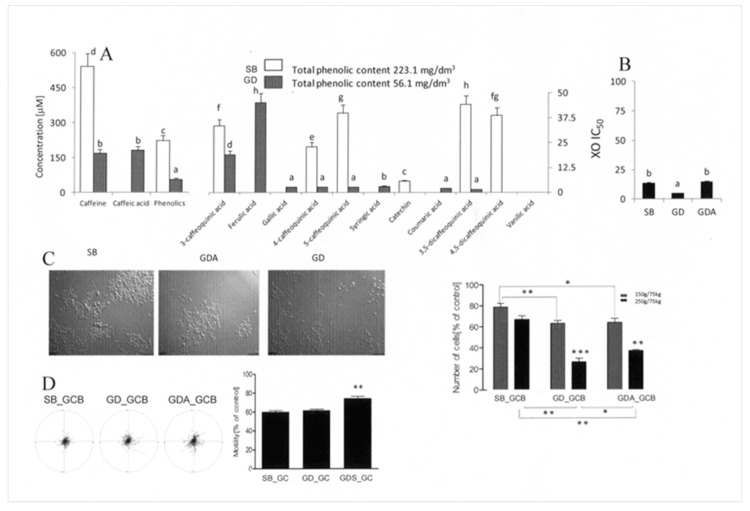
Gastrointestinal fate of compounds from GCB flour. (**A**) GCB were subjected to simulated mastication (SB) and gastrointestinal digestion (GD) in vitro and the extract was applied to culture medium at a concentration equivalent to 250 g of product per 75 kg of body weight. The bar graphs show theoretical medium concentrations of the compounds calculated on the basis of their content in the extract. (**B**) GCB were processed as above, and the antioxidative and XO inhibitory activity of the extracts was estimated. (**C**) DU145 cells were seeded at a density of 10^4^/cm^2^, and cell proliferation was measured after 72 h of incubation using a Coulter counter and compared to the control (= 100%). Scale bar: 100 μm. (**D**) DU145 cells were seeded at a density of 10^4^/cm^2^, cultivated for 24 h, and treated with the relevant extract for 72 h, and cell motility was analyzed via time-lapse videomicroscopy. Cell trajectories are presented in circular diagrams with the starting point of each trajectory situated at the plot center (*N* = 50). The bar graph shows averaged cell motility. All results are representative of at least three independent experiments performed in triplicate (*N* > 9). The values designated by the different letters (A and B) are statistically significantly different. *p*-values in C and D were calculated using the non-parametric Mann–Whitney test; *p ≤ 0.05, **p ≤ 0.01, ***p ≤ 0.001. Note the relatively low bioactivity and high phenolic content of the SB GCB extract.

To further investigate the effect of WMWB compounds on the nutritional potential of fortified WMWB, we assessed the “gastrointestinal fate” of bioactive GCB compounds. Masticated (SB) GCB extract contained an abundant fraction of phenolics (Figure 4A). It was also relatively rich in xanthine oxidase inhibitors (Figure 4B) and exerted a strong anti-invasive effect on DU145 cells (Figure 4D). On the other hand, its cytostatic activity was remarkably low (Figure 4C), which may confirm the presence of cytoprotective compounds in GCB. Gastrointestinal processing considerably reduced the total amounts of phenolics, caffeine, catechin, and caffeolquinic acids in GCB extract while releasing considerable amounts of antioxidants and caffeic and ferulic acids (Figure 4A). These shifts in phenolic content did not affect the strong inhibitory effect of GCB extract on the motility of DU145 cells. In turn, they were accompanied by increased cytostatic activity of the extract (Figure 4D). These observations confirm that the cytostatic activity of GCB compounds is retained in GCB extract after gastrointestinal processing. In addition, differences in proliferation- and motility-related DU145 susceptibility to the SB, GD, and GDA extracts show that the signaling pathways involved in both processes are differentially affected by their compounds. High cytoprotective activity of the hydrophilic fraction of GCB compounds may account for the relatively low bioactivity of the SB GCB extract. Due to their relatively low bioaccessibility, they no longer counterbalance the activity of cytostatic GCB compounds in GD extracts. Gastrointestinal digestion may also attenuate the activity of cytoprotective GCB compounds in the fortified product. On the other hand, the cytoprotective activity of WMWB compounds, which counteracts the cytostatic properties of their GCB counterparts, explains the surprisingly low bioactivity of GCB-fortified WMWB. Bioaccessible WMWB compounds can negatively affect the cytostatic activity of GCB phenolics in the fortified bread, even though their interference with the activity of cytostatic WMWB compounds is negligible. 

### 3.5. Prostate Cells Differ in Their Reactivity to Cytostatic/Cytoprotective WMWB/GCB Compounds 

**Figure 5:**
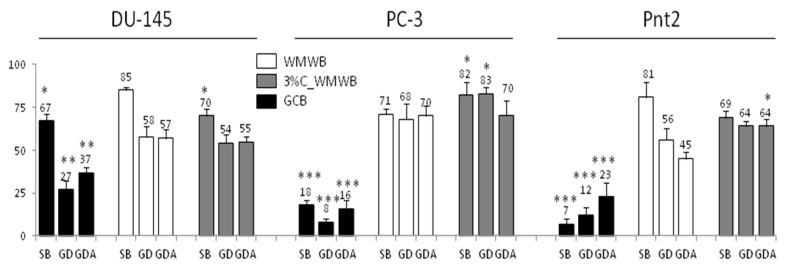
Cytostatic effects of GCB, WMWB, and its GCB-fortified variant on PC3 and Pnt2 cells. Cells were seeded at a density of 10^4^/cm^2^ and cultivated for 24 h before the extracts from GCB, WMWB, and its fortified variant were administered along with 10% fetal bovine serum (FBS)-supplemented culture medium. Cell proliferation was measured after 72 h of incubation using a Coulter counter and compared to the control (= 100%). All results are representative of at least three independent experiments performed in triplicate (*N* > 9). *p*-values were calculated against the relevant WMWB control using the non-parametric Mann–Whitney test; *p ≤ 0.05, **p ≤ 0.01, ***p ≤ 0.001. Note that GCB fortification attenuates the cytostatic activity of WMWB compounds against PC3 and Pnt2 cells.

Further analyses were performed to estimate how interactions between WMWB and GCB compounds and their gastrointestinal fate affect the magnitude of their cytostatic effects on human prostate cancer PC-3 and epithelial Pnt2 cells. In contrast to DU145 cells, which were propagated from prostate tumor metastasis to the brain, PC3 cells represent an aggressive cell line derived from bone metastases of human prostate tumors, whereas epithelial prostate Pnt2 cells display a non-transformed phenotype. Both PC-3 and Pnt2 cells displayed a high susceptibility to GCB extracts, which confirms the nutritional and chemopreventive potential of GCB. Notably, hydrophilic and bioaccessible compounds from the GCB-fortified WMWB exerted a less pronounced cytostatic effect on PC-3 and Pnt2 cells than their WMWB counterparts (Figure 5). Furthermore, both PC-3 and Pnt2 cells showed relatively low sensitivity to GD and GDA extracts from the fortified bread. These data show that prostate cells differ in the reactivity to cytostatic/cytoprotective WMWB/GCB compounds. They also confirm the importance of nonlinear effects of gastrointestinal processing in terms of the cytostatic activity of GCB/WMWB phenolics. Consequently, the cytoprotective activities of GCB/WMWB compounds should be taken into account when considering the pro-healthy effects of WMWB fortification and its significance for prostate cancer prevention and treatment.

Targeted and market-driven food fortification leads to the design of new products that meet the criteria of so-called ‘‘functional foods” [27,28]. Food fortification is usually aimed at increasing the content of health-promoting components in widely consumed products (e.g., bakery products, pastas, juices). Prospectively, this strategy is expected to prevent the deficiency of pro-healthy compounds resulting from commercial food processing [29,30] and to reduce the incidence of “civilization diseases”, including cancer. Here, we have confirmed that GCB contain numerous substances that can be used for prostate cancer chemoprevention. However, our observations also demonstrate that gastrointestinal processing of GCB-fortified bread foods may result in shifts in the WMWB/GCB compound concentrations. These may have profound consequences for the bioactivity of the product; in particular, they can account for nonlinear correlation between the cytostatic activity of the product and its phenolic content [31,32]. 

The interactions between WMWB and GCB compounds, which negatively affect the bioactivity of GCB-fortified WMWB, may have chemical, physical, and physiologic backgrounds. At the physico-chemical level, direct interactions between the compounds may be responsible for nonlinear associations between their concentrations and activity in complex mixtures. These associations lead to reciprocal synergistic/antagonistic interactions between the compounds of the fortifier and the carrier that can be translated into differences in their bioactivity and bias the interpretation of in vitro analyses [31,32]. Concomitantly, the physical properties of cytoprotective compounds (resulting in matrix cross-linking and precipitation) affect their bioavailability in the specific cell microenvironment. Changes in this parameter may also result from the interactions of GCB and WMWB phenolics with gelatinized starch granules and the gluten network of the bread. Bread preparation can additionally promote the formation of protein cross-links, hydrophobic and ionic interactions, and hydrogen and covalent bonding between the matrix and phenolics. Additionally, interactions between phenolics with salivary proline-rich proteins and/or other digestive enzymes and food proteins can differentially affect the bioaccessibility of cytoprotective and cytostatic WMWB/GCB compounds [24,33]. 

At the physiological level, food processing, digestion, and serum interactions determine the final biological effect of WMWB fortification, whereas intrinsic cellular systems are crucial for the susceptibility and adaptation of cancer cells to cytostatic stress. The bioactivity of the fortified product may also be affected by the momentary physiological status of the whole organism, its multicellular systems, and single cells. Concomitantly, the quality and quantity of gastrointestinal processing is determined by the physical status of the organism. Furthermore, secular and permanent cellular phenotypes, in particular, the activity of cellular detoxification systems, is crucial for cell reactivity to individual compounds and to their mixtures [34,35]. Prostate cancer cells display relatively high phenotypic heterogeneity. This is manifested by differences in the activity of intracellular signaling pathways in discrete prostate cancer cell lineages and leads to their different susceptibility levels to the cytostatic/chemopreventive action of administered compounds. In our hands, the cytoprotective effect of WMWB compounds prevailed over their possible cytostatic activity in the PC-3 model, which was not the case for DU145 cells. At the cellular level, the activity of multi-drug-resistant (MDR) systems can affect the intracellular bioavailability of the compounds. Most importantly, the cytostatic/cytotoxic properties of phenolic compounds are often interspersed with their cytoprotective activities, and the final effect is highly dependent on cellular context. 

For instance, nutritional antioxidants can perform as synergists to reduce ROS via quenching free radicals in both their aqueous and lipid phases. Thus, they preserve the genomic stability of cells [36]. However, ROS are also involved in the physiological regulation of the signaling pathways which determine cancer cell proliferation and motility [37]. Therefore, the final activity of antioxidants can vary between growth inhibition and cytoprotection. This is illustrated by the activity of xanthine oxidase (XO), the products of which act as regulators of cancer cell proliferation and migration [37,38]. Therefore, the XO-inhibiting activity of the extract may add to its overall cytostatic activity. However, phenolics can also act as scavengers of pro-oxidative XO products (for instance, superoxides) [39], thus exerting cytoprotective effects on cancer cells.

## 4. Conclusions

Collectively, we showed that the complex interplay between the cytostatic and cytoprotective activities of WMWB and GCB compounds may lessen the pro-healthy activity of GCB-fortified WMWB at the cellular level. This indicates that the application of GCB for WMWB fortification should be considered with caution. Further studies are necessary to elucidate the mechanism(s) underlying the specificity of WMWB/GCB effects on prostate cancer and normal cell physiology. They should also focus on the interactions between individual GCB and WMWB compounds. These issues should be addressed before the fortification of a specific carrier with a specific supplement is recommended [40].
